# Methodological insights regarding the prognostic value of lncRNA PGM5P4-AS1 in breast cancer

**DOI:** 10.1080/15384047.2026.2614802

**Published:** 2026-01-13

**Authors:** Rashad Ismayilov, Arzu Oguz

**Affiliations:** a Department of Medical Oncology, Baskent University Faculty of Medicine, Ankara, Türkiye

To the Editor,

We read with great interest the recent article by Wu et al., “Downregulation of the lncRNA PGM5P4-AS1 predicts poor prognosis and drives breast cancer progression through miR-3664-5p/KLF9.”^1^ The authors present compelling evidence identifying PGM5P4-AS1 as a potential prognostic biomarker. However, we wish to raise three methodological and theoretical points that merit further discussion to clarify the clinical and biological implications of these findings.

First, regarding statistical methodology, the authors performed a post–hoc power analysis to justify their sample size, calculating a power of 87.7% based on the observed hazard ratio. As established by Hoenig and Heisey, calculating power using observed effect sizes after data analysis is statistically invalid, often termed the “power approach paradox.”^2^ Once the study is complete, the confidence interval provides the necessary information regarding precision; retrospective power calculations provide no additional insight and are analytically circular.

Second, the study relies on the competing endogenous RNA (ceRNA) hypothesis, positing that PGM5P4-AS1 acts as a “sponge” for miR-3664-5p. While the authors demonstrate binding via luciferase assays, the plausibility of the ceRNA effect depends heavily on stoichiometry. For a lncRNA to effectively sequester a miRNA, its cellular abundance must be comparable to or exceed that of the miRNA target pool.^3^ Cytoplasmic lncRNAs are often expressed at low copy numbers (<1 copy/cell), whereas miRNAs can exist in the thousands. Without absolute quantification of PGM5P4-AS1 and miR-3664-5p copy numbers, it remains unclear if the lncRNA is abundant enough to drive the observed phenotypic changes solely through miRNA sponging.

Finally, we noted a potential confounding issue in the clinical survival analysis. Figure 2 A indicates that PGM5P4-AS1 expression is significantly lower in triple–negative breast cancer (TNBC) and HER2+ subtypes compared to Luminal A. Historically, intrinsic subtypes are potent determinants of survival.^4^ Surprisingly, in the authors' multivariate Cox regression (Table 2), the “Subtypes” variable was not statistically significant (*p* = 0.286). Given the well–established survival disparities between subtypes, this loss of significance suggests potential multicollinearity between the lncRNA expression and the subtype. It raises the question of whether PGM5P4-AS1 is an independent driver or a surrogate marker for the aggressive TNBC phenotype.

We commend the authors for this extensive work and believe that addressing these stoichiometric and statistical nuances will further strengthen the utility of PGM5P4-AS1 as a biomarker in breast oncology.

## Data Availability

There is no data associated with this research.
